# Acute kidney injury among hospitalised patients who died due to COVID-19 in the Eastern Cape, South Africa

**DOI:** 10.4102/safp.v65i1.5616

**Published:** 2023-01-13

**Authors:** Ramprakash Kaswa

**Affiliations:** 1 Department of Family Medicine and Rural Health, Faculty of Health Sciences, Walter Sisulu University, Mthatha, South Africa

**Keywords:** AKI, COVID-19, hospitalised, comorbidity, prognosis

## Abstract

**Background:**

Acute kidney injury (AKI) commonly occurs in coronavirus disease 2019 (COVID-19) patients who have been hospitalised and is associated with a poor prognosis. This study aimed to determine the incidence of AKI among COVID-19 patients who died in a regional hospital in South Africa.

**Methods:**

This retrospective record review was conducted at the Mthatha Regional Hospital in South Africa’s Eastern Cape province. Data were collected between 10 July 2020 and 31 January 2021.

**Results:**

The incidence of AKI was 38% among the hospitalised patients who died due to COVID-19. Most study participants were female, with a mean age of 63.3 ± 16 years. The most common symptom of COVID-19 at the time of hospitalisation was shortness of breath, followed by fever and cough. Half of the patients had hypertension, while diabetes, human immunodeficiency viruses (HIV) and tuberculosis (TB) were other comorbidities. At admission, the average oxygen saturation was 75.5% ± 17.

**Conclusion:**

The study revealed a high incidence of AKI among hospitalised patients who died due to COVID-19. It also found that those received adequate crystalloid fluids at the time of admission had a lower incidence of AKI.

**Contribution:**

Acute kidney injury can be prevented by adequate fluid management during early stage of COVID-19. Majority of COVID-19 patients were referred from lower level of care and primary care providers have their first encounter with these patients. Adequate fluid resuscitation in primary care settings can improve the outcome of hospitalised COVID-19 patients.

## Introduction

The rapid progression of the severe acute respiratory syndrome coronavirus 2 (SARS-CoV-2) pandemic is a global challenge.^1,2^ The new evidence of coronavirus disease 2019 (COVID-19) is constantly closing the gap between pathogenesis and variability in the clinical presentation.^3,4^ Coronavirus disease 2019 presented in a wide range from asymptomatic to severe forms of the disease with multisystem failure.^5,6^ The respiratory system involvement is the main feature of COVID-19 and lung involvement in the form of severe viral pneumonia characterised by a massive inflammatory infiltrate and endothelial damage resulting in acute respiratory distress syndrome (ARDS).^1,7^ The full spectrum of SARS-CoV-2 impact on other organs, including the kidney, is not well understood.^2,8^

The new evidence is emerging with variable kidney involvement and substantial geographic difference.^2,6^ The kidney involvement has not been well understood, but acute kidney injury (AKI) in COVID-19 patients is of great concern.^9^ There are various theories regarding the cause of AKI in patients with COVID-19 infection. It is believed that the virus-mediated injury and the dysregulation of the angiotensin II pathway are the factors that trigger this condition. However, the exact management of AKI in these patients remains unclear.^10,11^

The incidence of AKI among COVID-19 hospitalised patients has ranged from 5% to 29%, with high mortality.^6,12,13^ The clinical course of kidney involvement is utterly unpredictable, from asymptomatic infection to multi-organ system failure.^11,14^ The mortality rate among patients with COVID-19 with superimposed AKI remains exceptionally high.^15,16^ There is a need to understand the different clinical presentations of COVID-19 and its impact on clinical outcomes. There is currently no evidence supporting the idea that the management of COVID-19 associated AKI should be different from other causes of AKI.

A few studies from the United States, Europe and China described the characteristics and outcomes of AKI among COVID-19 hospitalised patients.^12,17,18^ The evidence of COVID-19-associated AKI and its effect in South Africa have been limited.^8^ The incidence of AKI among people with COVID 19 substantially varies between different populations and geographic regions.^2,6^ The specific effect of SARS-CoV-2 on the kidney is not yet fully understood and to what extent it increases the risk of AKI.^8,19,20^ More evidence on clinical characteristics and outcomes of COVID-19-associated AKI is needed to optimise the management and prevention of these complications.

Understanding the epidemiology of COVID-19 in patients with different AKI stages is vital to identifying potential risk factors and improving the clinical management of this disease. Current literature suggests that the kidney is vulnerable in COVID-19 patients, but there is a lack of available data regarding its incidence and outcomes in South Africa.^8,14^ This study provides insight into the planning and resources needed for the subsequent phases of the pandemic. The study aimed to determine the incidence of AKI in a hospitalised patient who died due to COVID-19 in a regional hospital in the Eastern Cape province of South Africa.

## Methods

### Study design and settings

Retrospective record review of the first 100 patients who died due to COVID-19 between 10 July 2020 and 31 January 2021 was conducted at Mthatha Regional Hospital (MRH) in the Eastern Cape province of South Africa. Patients were admitted to an isolation ward after confirming a positive result of reverse-transcriptase polymerase chain reaction (RT-PCR) or rapid antigen test for SARS-CoV2 virus from a nasopharyngeal swab. Mthatha Regional Hospital is a 302-bed referral hospital designated for COVID-19 management. The hospital has a 36-bed isolation unit to accommodate COVID-19 patients, and additional beds were repurposed during the pandemic’s peak to accommodate the other COVID-19 patients. The hospital provides level one and two care to approximately half a million people in King Sabata Dalindyebo (KSD) sub-district municipality.

King Sabata Dalindyebo is one of the rural municipalities in the Eastern Cape province of South Africa. The community heavily depends on social welfare grants, with about a 35% unemployment rate. About 4.5% of the population can afford private medical insurance, and most people utilised state health facilities for healthcare needs.^21^

Data were manually collected from clinical health records by a trained research assistant and included demographic characteristics, comorbid conditions and AKI. Comorbid conditions derived from the patients were abstracted from the documentation on the clinical health records. An Excel spreadsheet was used to extract the data, and every 10th entry was rechecked by the researcher for accuracy and quality assurance.

### Definition of acute kidney injury

The current study adopted the criteria of the Kidney Disease Improving Global Outcome (KDIGO) and International Society of Nephrology guidelines to classify the case definition of AKI. The AKI was defined as an increase in serum creatinine level more than 1.5 times from baseline value within a week or an increase in the absolute values of serum creatinine by 26.5 µmol/L within 48 h.^20^

Most of the patients did not have preadmission baseline creatinine values; therefore, the first creatinine value recorded during admission was considered baseline. The study did not include urine output to determine the case definition of AKI. Patients with end-stage renal disease and only one reading of serum creative before they died were excluded from the study.

### Outcome measures and analyses

Demographic factors (age, gender and employment status) and significant comorbid conditions (diabetes, hypertension, diabetes and cardio-respiratory diseases) were extracted from clinical records. In addition, baseline data of vital signs and oxygen mode were recorded. The study describes the mean, standard deviation for continuous variables and frequency and proportion for categorical variables as descriptive statistics. Chi-squared test was used to analyse the associations between demographic factors, comorbid conditions and AKI incidence, and a two-sided *p* < 0.05 was considered statistically significant. Data were analysed using Statistical Package for Social Sciences (SPSS) version 18.0.

### Ethical considerations

The study was carried out under the Declaration of Helsinki and was approved by the Human Research and Ethics Committee of the Walter Sisulu University (reference number: 098/2020). In addition, the Eastern Cape Department of Health (reference number: EC_202010_027) and hospital management also approved this study.

## Results

The study analysed the clinical records of first 100 COVID-19 deaths at MRH during the study periods. The majority were female (57%) with a mean age of 63.3 ± 16 years. The incidence of AKI was 38% among hospitalised patients who died due to COVID-19. Shortness of breath was the most common presenting symptom of COVID-19, followed by weakness, cough and fever. Half of the hospitalised patients who died from COVID-19 had hypertension (50%), followed by diabetes mellitus (37%), HIV (15%), and TB (11%) comorbidity. About one third (34%) of patients had more than one comorbidity and majority of them had diabetes and hypertension coexist. The mean oxygen saturation (SpO_2_) at admission was 75.5 ± 17 on pulse oximetry. The majority of the patient received supplementary oxygen by facemask (66%) followed by nasal high flow (28%), continuous positive airway pressure (CPAP) (3%) and 3% were on room air. The demographic and clinical characteristics of hospitalised patients who died due to COVID-19 are demonstrated in Table 1.

**TABLE 1:** Clinical characteristics of the hospitalised patient who died due to COVID-19.

Characteristics	Number
**Gender (*n* = 100)**	
Male	43
Female	57
**Presenting symptoms (*n* = 100)**	
Shortness of breath	91
Weakness	87
Cough	68
Fever	33
Loss of test	7
Loss of smell	4
**Comorbidity (*n* = 100)**	
Hypertension	50
Diabetes	37
HIV	15
TB	11
CVA	5
CKD	4
COPD	3
Epilepsy	3
**Supplementary oxygen methods (*n* = 100)**	
Face mask	66
Nasal high flow	28
CPAP	3
Room air	3

Note: Mean age ± s.d. 63.3 ± 16.

TB, tuberculosis; HIV, human immunodeficiency virus; CVA, cerebrovascular accident; CKD, chronic kidney disease; COPD, chronic obstructive pulmonary disease; CPAP, continuous positive airway pressure.

About two-thirds (60%) of hospitalised patients who died due to COVID-19 illness were above 60 years, and 13% were above 80 years of age. Table 2 demonstrates the age distribution across the gender, and most of those above 80 years were female.

**TABLE 2:** Age and gender distribution of hospitalised patients who died due to COVID-19.

Age (years)	Male	%	Female	%	Total	%
≤ 20	1	2.3	0	0.0	1	1
21–40	2	4.7	9	15.8	11	11
41–60	11	25.6	17	29.8	28	28
61–80	27	62.8	20	35.1	47	47
> 80	2	4.7	11	19.3	13	13

**FIGURE 1:**
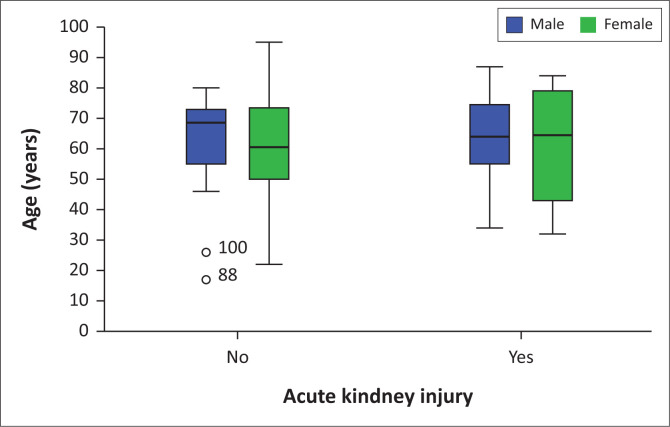
Age, gender and acute kidney injury distribution among hospitalised patients who died due to COVID-19.

Figure 1 demonstrates the relationship between age and AKI among hospitalised people who died due to COVID-19. There was no significant age difference between AKI and non-AKI groups in both males and females.

**FIGURE 2:**
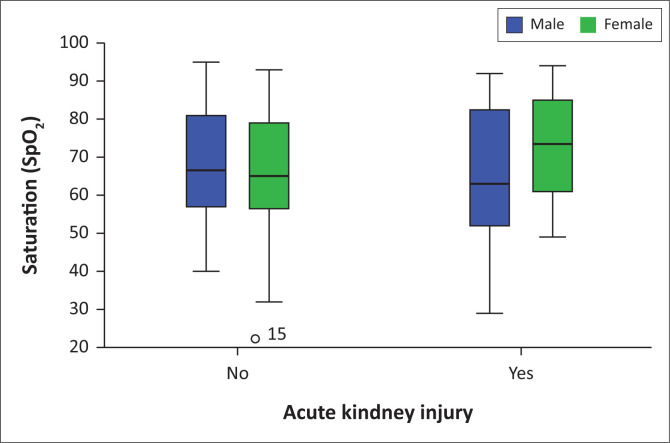
Oxygen saturation, gender and acute kidney injury distribution among hospitalised patients who died due to COVID-19.

There was no significant difference between mean oxygen saturation by pulse oximetry at the time of admission in the non-AKI group. However, further gender analysis among AKI group male patients has lower mean saturation at the time of admission than their non-AKI counterparts, but the difference was not statistical significant. Figure 2 demonstrates the relationship between oxygen saturation and AKI among male and female hospitalised patients who died due to COVID-19.

**FIGURE 3:**
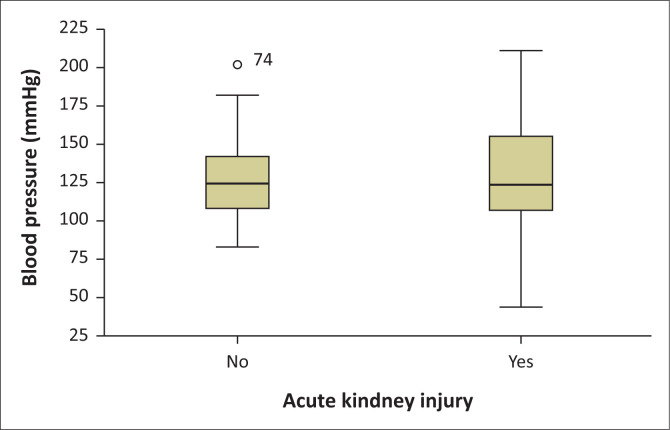
Mean blood pressure, gender and acute kidney injury distribution among hospitalised patients who died due to COVID-19.

There was no significant association between mean blood pressure and AKI among hospitalised patients who died due to COVID-19, except that the AKI group had a wide blood pressure distribution. Figure 3 demonstrates the distribution of blood pressure between AKI and non-AKI groups.

**FIGURE 4:**
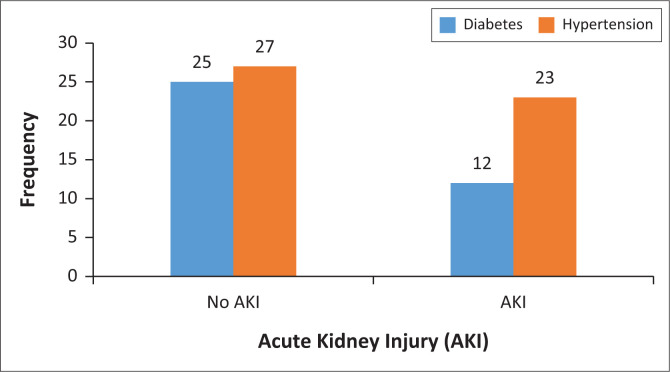
Comorbidity and acute kidney injury distribution among hospitalised patients who died due to COVID-19.

Only one-third (32%) of diabetic patients developed AKI, whereas about half (46%) of hypertensive patients had AKI who died due to COVID-19. There was a negative correlation between diabetes control and AKI among hospitalised COVID-19 patients. Figure 4 demonstrates the incidence of AKI among COVID-19 patients with comorbidity of diabetes and hypertension.

**FIGURE 5:**
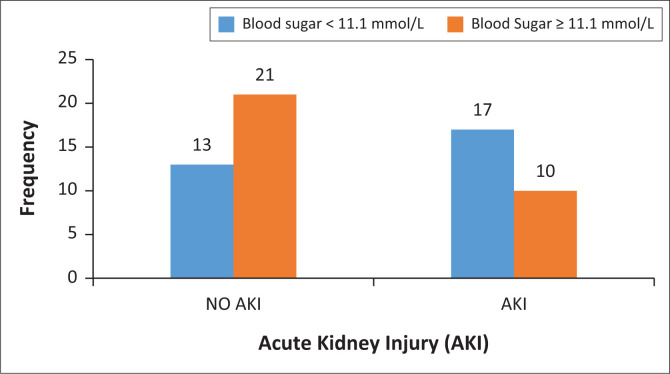
Blood glucose and acute kidney injury distribution among hospitalised patients who died due to COVID-19.

Coronavirus disease 2019 patients with high blood sugar had a lower risk of AKI than those with normal blood sugar. A statistically significant difference was found between AKI and serum blood sugar at admission (χ^2^ = 3.2, *p* = 0.04). Patient who presented with blood sugar > 11.1 mmol/L at the time of admission had lower incidence of AKI compare to their counterpart who presented with blood sugar < 11.1 mmol/L. Figure 5 demonstrates the relationship between AKI and serum blood sugar level at the time of admission among hospitalised patients who died due to COVID-19.

## Discussion

This study identified a significant proportion of AKI among hospitalised COVID-19 patients in a regional hospital. The incidence of AKI was 38% among hospitalised patients who died due to COVID-19. Several studies reported the incidence of AKI was associated with an increased mortality rate within 30 days following admission.^6,9,12^ Compared with patients who did not have AKI, those with AKI were more likely to experience a worse prognosis.^6^ Acute kidney injury was an independent risk factor of COVID-19 mortality among hospitalised patients.^1^ The link between poor prognosis and AKI has been well established by several studies during the COVID-19 pandemic.^1,9^ Although our study’s findings did not establish the causal relationship between AKI and mortality but high incidence of AKI among COVID-19 patients was consistent with current literature findings. The high AKI rates in these patients are consistent with the large United States patient population study (32% – 46%) and United Kingdom cohort study (39%).^9,11^ A study by Chen et al. revealed that AKI was prevalent in 30% of the hospitalised patients, and it increased to more than 45% in those requiring admission to the ICU.^7^ The various factors such as the quality of healthcare and the cost and availability of kidney replacement therapy in different countries are also known to have a significant impact.^14^

The most common comorbidities reported in this study were hypertension, diabetes mellitus, HIV and pulmonary tuberculosis (TB). The results of this study suggest that diabetes and hypertension are independent factors that can increase the risk of AKI among COVID-19 hospitalised patients. Several studies demonstrated that diabetes and hypertension were associated with an increased AKI rate among COVID-19 hospitalised patients.^7,9^ The current evidence supports the link between these conditions and the development of AKI in COVID-19 patients.^18^ It has been reported that multiple factors such as smoking, high body mass index (BMI) and diabetes are known to increase the risk of developing AKI.^9^

Interestingly, there was no link between the mean age, blood pressure and oxygen saturation at the time of admission and the development of AKI among COVID-19 hospitalised patients. It was possible that these factors could aggravate the risk of COVID-19 mortality. On the contrary, a study from two tertiary centres in South Africa reported that AKI patients were older and more likely to have pre-existing hypertension.^8^ Age has not been shown as a risk factor for the development and severity of COVID-19-related AKI in our study. The statistical difference between the two groups was not significant.

Due to the emergence of multiple mechanisms that can cause AKI, non-dialytic management is becoming more critical in delaying the start of renal replacement therapy.^5^ The lack of resources during the pandemic has severely affected the treatment of AKI.^8^ In addition, patients who received more than 2 L of crystalloid fluids during hospital admission had a lower incidence of AKI. During the pandemic, there was a concern that COVID-19 patients might develop a capillary fluid leak that could lead to a clinical condition known as a hyper-inflammatory state.^8,17^ This concern prompted many clinicians to be cautious about performing the excess fluid replacement.^5^ However, as the COVID-19 pandemic progressed, the clinical community became more aware of the increasing volume depletion and AKI rates. In addition, the clinician became more experienced in managing these patients. As a result, it shifted towards a more liberal, fluid strategy.^5,8^

Acute kidney injury increases the risk of death in critically ill patients, such as COVID-19. It is also known to have a poor prognosis even if the initial COVID-19 severity is not severe enough to trigger kidney involvement.^8,19^ Early detection and treatment of renal dysfunction can improve the prognosis of COVID-19 patients.^17^ The study findings highlighted the importance of increasing awareness regarding the seriousness of AKI prevention among COVID-19 patients. In addition, these findings recommend specific treatment approaches such as judicious fluid management in COVID-19 patients that can prevent AKI-induced mortality.

### Limitation of the study

The data collected from this study were limited to a single public health facility. Therefore, the findings of the study could not be generalised. Also, the data are only limited to patient-specific observations. This means that a healthcare professional performs the diagnosis and assessment of patients. This study did not include the patients who recovered from COVID-19 and had AKI during hospitalisation. Also, the study did not consider the clinical parameters affecting the disease’s severity. Therefore, the study findings cannot distinguish deaths from COVID-19 or underlying AKI or other medical conditions.

## Conclusion

The study revealed that AKI was common among hospitalised patients who died due to COVID-19 in a regional hospital setting. This condition can be prevented during the early stage of COVID-19 infection in a resource-poor setting through adequate fluid resuscitation. Further research is needed to explain various factors that can affect the development and prognosis of AKI in COVID-19 patients.

## References

[1] Hansrivijit P, Qian C, Boonpheng B, et al. Incidence of acute kidney injury and its association with mortality in patients with COVID-19: A meta-analysis. J Investig Med. 2020;68(7):1261–1270. 10.1136/jim-2020-001407 32655013

[2] Brienza N, Puntillo F, Romagnoli S, Tritapepe L. Acute kidney injury in coronavirus disease 2019 infected patients: A meta-analytic study. Blood Purif. 2021;50(1):35–41. 10.1159/000509274 32615555 PMC7445379

[3] Batlle D, Soler MJ, Sparks MA, et al. Acute kidney injury in COVID-19: Emerging evidence of a distinct pathophysiology. J Am Soc Nephrol. 2020;31(7):1380–1383. 10.1681/ASN.2020040419 32366514 PMC7350999

[4] Gabarre P, Dumas G, Dupont T, Darmon M, Azoulay E, Zafrani L. Acute kidney injury in critically ill patients with COVID-19. Intensive Care Med. 2020;46(7):1339–1348. 10.1007/s00134-020-06153-9 32533197 PMC7290076

[5] Huang C, Wang Y, Li X, et al. Clinical features of patients infected with 2019 novel coronavirus in Wuhan, China. Lancet. 2020;395(10223):497–506. 10.1016/S0140-6736(20)30183-5 31986264 PMC7159299

[6] Raina R, Mahajan ZA, Vasistha P, et al. Incidence and outcomes of acute kidney injury in COVID-19: A systematic review. Blood Purif. 2022;51:199–212. 10.1159/000514940 34130296 PMC8339045

[7] Chen Y-T, Shao S-C, Hsu C-K, Wu I-W, Hung M-J, Chen Y-C. Incidence of acute kidney injury in COVID-19 infection: A systematic review and meta-analysis. Crit Care. 2020;24(1):346. 10.1186/s13054-020-03009-y 32546191 PMC7296284

[8] Diana NE, Kalla IS, Wearne N, et al. Acute kidney injury during the COVID-19 pandemic – Experience from two tertiary centres in South Africa. Wits J Clin Med. 2020;2(3):137. 10.18772/26180197.2020.v2n3a2

[9] Nimkar A, Naaraayan A, Hasan A, et al. Incidence and risk factors for acute kidney injury and its effect on mortality in patients hospitalized from COVID-19. Mayo Clin Proc Innov Qual Outcomes. 2020;4(6):687–695. 10.1016/j.mayocpiqo.2020.07.003 32838205 PMC7368916

[10] Kooman JP, Van der Sande FM. COVID-19 in ESRD and acute kidney injury. Blood Purif. 2021;50(4–5):610–620. 10.1159/000513214 33321496 PMC7802200

[11] Chan L, Chaudhary K, Saha A, et al. AKI in hospitalized patients with COVID-19. J Am Soc Nephrol. 2021;32(1):151–160. 10.1681/ASN.2020050615 32883700 PMC7894657

[12] Bowe B, Cai M, Xie Y, Gibson AK, Maddukuri G, Al-Aly Z. Acute kidney injury in a national cohort of hospitalized US veterans with COVID-19. Clin J Am Soc Nephrol. 2021;16(1):14–25. 10.2215/CJN.09610620 PMC7792643 33199414

[13] Paek JH, Kim Y, Park WY, et al. Severe acute kidney injury in COVID-19 patients is associated with in-hospital mortality. PLoS One. 2020;15(12):1–12. 10.1371/journal.pone.0243528 PMC7725289 33296419

[14] Walther CP, Podoll AS, Finkel KW. Summary of clinical practice guidelines for acute kidney injury. Hosp Pract. 2014;42(1):7–14. 10.3810/hp.2014.02.1086 24566591

[15] Ali H, Daoud A, Mohamed MM, et al. Survival rate in acute kidney injury superimposed COVID-19 patients: A systematic review and meta-analysis. Ren Fail. 2020;42(1):393–397. 10.1080/0886022X.2020.1756323 32340507 PMC7241495

[16] VAN HOUGENHOUCK-TULLEKEN W, Hussain M, do Vale C. POS-062 Acute kidney injury in COVID-19 infection: What are the outcomes in South Africa?. Kidney Int Reports. 2021;6(4):S27–S28. 10.1016/j.ekir.2021.03.068

[17] Hirsch JS, Ng JH, Ross DW, et al. Acute kidney injury in patients hospitalized with COVID-19. Kidney Int. 2020;98(1):209–218. 10.1016/j.kint.2020.05.006 32416116 PMC7229463

[18] Kolhe NV, Fluck RJ, Selby NM, Taal MW. Acute kidney injury associated with COVID-19: A retrospective cohort study. PLoS Med. 2020;17(10):1–16. 10.1371/journal.pmed.1003406 PMC7598516 33125416

[19] Chueh T-I, Zheng C-M, Hou Y-C, Lu K-C. Novel evidence of acute kidney injury in COVID-19. J Clin Med. 2020;9(11):3547. 10.3390/jcm9113547 33153216 PMC7692179

[20] Rudd KE, Cizmeci EA, Galli GM, Lundeg G, Schultz MJ, Papali A. Pragmatic recommendations for the prevention and treatment of acute kidney injury in patients with COVID-19 in low- and middle-income countries. Am J Trop Med Hyg. 2021;104(3):87–96. 10.4269/ajtmh.20-1242 33432912 PMC7957240

[21] KSD. King Sabata Dalindyebo Local Municipality – Basemap [homepage on the Internet]. 2022. [cited 2022 July 15] Available from https://www.saferspaces.org.za/uploads/files/APCOF_Municipal_SP_King_Sabata_WEB.pdf

